# Posterior Circulation Stroke as a Time-Sensitive Systems Failure: A Case of Basilar Artery Occlusion With Multisystem Complications

**DOI:** 10.7759/cureus.106837

**Published:** 2026-04-11

**Authors:** Alireza Izadian Bidgoli, Alberto Gomez Veliz, Amanda Pina, Gabriel A Saavedra, Guillermo Rame

**Affiliations:** 1 Medicine, American University of the Caribbean School of Medicine, Cupecoy, SXM; 2 Internal Medicine, Jackson Memorial Hospital, Miami, USA; 3 Medicine, Ross University School of Medicine, Two Mile Hill, BRB

**Keywords:** basilar artery occlusion, endovascular thrombectomy, multisystem complications, perfusion imaging mismatch, posterior circulation stroke

## Abstract

Posterior circulation stroke, particularly basilar artery occlusion, represents a neurologic emergency with high morbidity and mortality, often complicated by diagnostic ambiguity and rapid systemic deterioration. We present the case of a 56-year-old male with multiple vascular risk factors who presented with acute altered mental status, dysarthria, and focal neurologic deficits. Initial evaluation considered a broad differential diagnosis, including intracranial hemorrhage, seizure, and metabolic derangements; however, rapid multimodal assessment excluded these etiologies and supported an acute ischemic process. Advanced imaging demonstrated high-grade basilar artery stenosis with a large perfusion mismatch, indicating a substantial volume of salvageable tissue despite minimal early infarction. Based on these findings, intravenous thrombolysis was administered within the therapeutic window, followed by transfer for neurointerventional evaluation and successful endovascular reperfusion. The clinical course was complicated by aspiration pneumonia, acute kidney injury, and metabolic instability, reflecting the systemic impact of posterior circulation ischemia. Magnetic resonance imaging confirmed an acute cerebellar infarction without hemorrhagic transformation. With coordinated multidisciplinary care, the patient demonstrated gradual neurologic improvement and was discharged in stable condition with residual deficits. This case supports a systems-based conceptual framework for posterior circulation stroke as a time-sensitive process, in which outcomes are influenced not only by timely reperfusion but also by early diagnostic clarity and proactive management of secondary physiologic complications.

## Introduction

Posterior circulation ischemic stroke represents a clinically distinct subset of cerebrovascular disease, accounting for approximately 20% of all ischemic strokes and involving the vertebrobasilar arterial system [1]. Among these, basilar artery occlusion (BAO) is one of the most severe and life-threatening entities, associated with high rates of disability and mortality in the absence of timely reperfusion [1]. Unlike anterior circulation stroke, posterior circulation events frequently present with subtle, fluctuating, or non-localizing neurologic symptoms, including dizziness, dysarthria, ataxia, and altered mental status, which can delay recognition and complicate early diagnosis [1]. This clinical variability contributes to a higher risk of misdiagnosis or delayed diagnosis, particularly in early presentations when imaging findings may be minimal or inconclusive.

Advances in acute stroke care have shifted clinical decision-making from rigid time-based thresholds toward a physiology-driven approach. Contemporary guidelines emphasize the role of multimodal imaging, including computed tomography perfusion and diffusion weighted magnetic resonance imaging, in identifying salvageable brain tissue and guiding reperfusion strategies [2]. This paradigm is particularly relevant in posterior circulation stroke, where early imaging may underestimate ischemic burden, and clinical deterioration can be rapid and unpredictable [1,2].

More recently, randomized controlled trials have provided strong evidence supporting endovascular therapy in patients with BAO. The ATTENTION (Endovascular Treatment for Acute Basilar-Artery Occlusion) and BAOCHE (Basilar Artery Occlusion Chinese Endovascular) trials demonstrated improved functional outcomes with thrombectomy compared with medical management alone, even when performed beyond traditional treatment windows, reinforcing the importance of early recognition and timely intervention [3,4]. Importantly, restoration of vessel patency alone does not fully determine clinical outcomes.

Emerging evidence suggests that posterior circulation stroke, particularly when involving the brainstem, often evolves as a multisystem process. Brainstem dysfunction can impair airway protection and autonomic regulation, predisposing patients to complications such as aspiration and physiologic instability, which may independently worsen prognosis despite successful reperfusion [1,2].

In this context, posterior circulation stroke may be conceptualized as a time-sensitive systems-based process, as illustrated by this case. In this framework, “systems failure” reflects the convergence of multiple factors, including delays in clinical recognition due to variable presentation, in-hospital workflow challenges related to imaging and intervention, and evolving physiologic instability driven by brainstem dysfunction and systemic complications. By explicitly linking diagnostic uncertainty, care delivery processes, and downstream physiologic consequences, this framework provides a structured approach to understanding how outcomes in posterior circulation stroke are determined. We present a case of BAO complicated by multisystem involvement, highlighting how this systems-based perspective may inform clinical decision-making and multidisciplinary care.

## Case presentation

Clinical presentation

A 56-year-old male with a past medical history significant for type 2 diabetes mellitus, hypertension, and prior cerebrovascular accident (June 2023) with residual left-sided weakness was brought to the emergency department after being found on the floor by emergency medical services. The patient was last known well approximately 15 minutes prior to the 911 call, establishing a clearly defined time of symptom onset. On arrival, he was noted to be confused with dysarthria, left-sided motor deficits, and evidence of head contusion. Initial vital signs were notable for severe hypertension (blood pressure 213/119 mmHg), tachycardia (heart rate 114 bpm), and tachypnea (respiratory rate 29 breaths/min), with oxygen saturation of 100% on room air. Neurologic examination demonstrated an alert but confused patient with left-sided weakness and facial droop. The National Institutes of Health Stroke Scale (NIHSS) score on arrival was 8, consistent with a moderate stroke.

The combination of acute altered mental status, dysarthria, and focal motor deficits raised immediate concern for posterior circulation stroke, particularly basilar artery involvement, given the presence of non-localizing neurologic findings. A stroke alert was activated immediately upon presentation to expedite emergent imaging and evaluation for reperfusion therapy.

Diagnosis and workup

**Figure 1:**
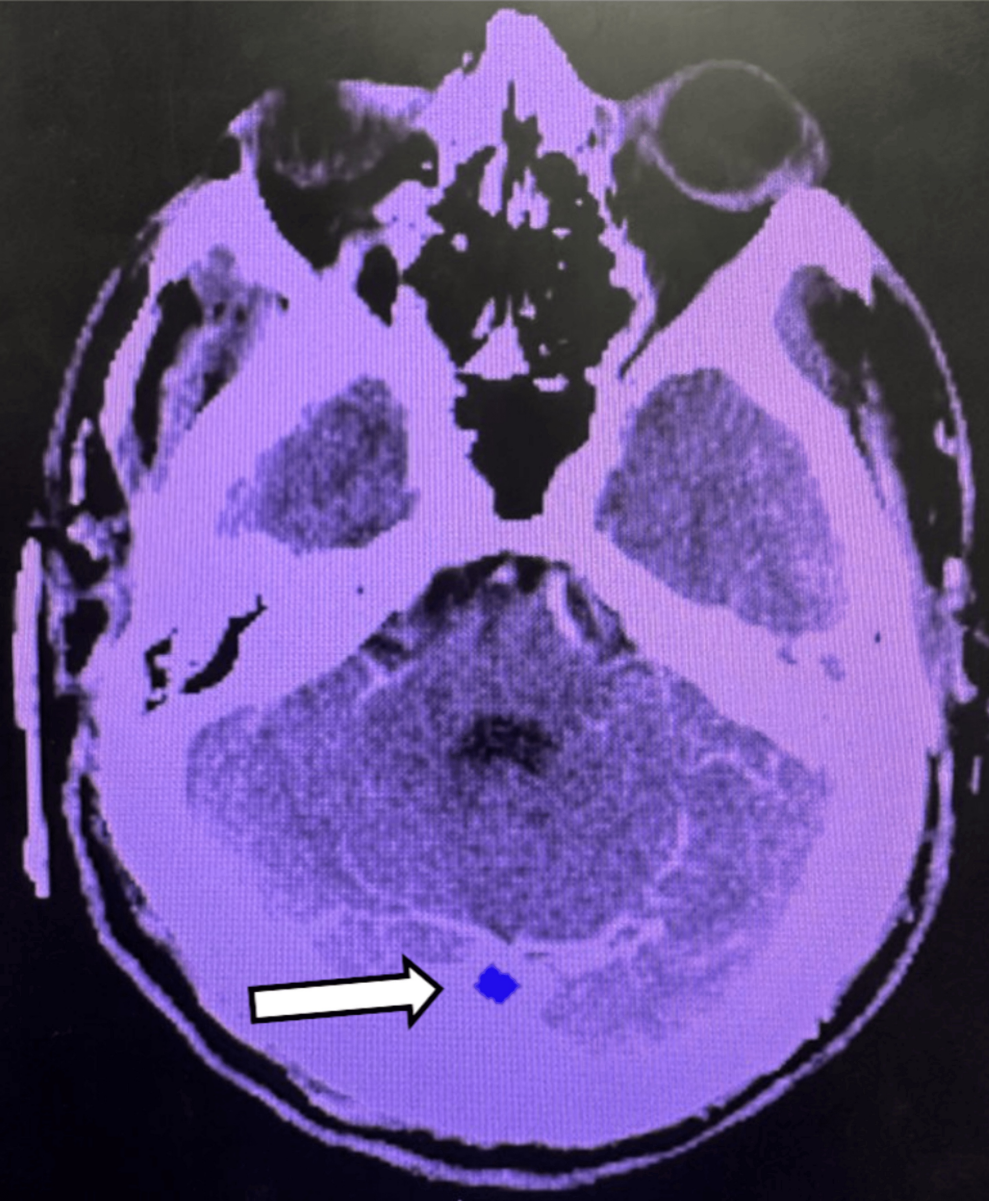
Non-contrast CT head demonstrating early posterior circulation ischemia. Axial non-contrast CT image demonstrates subtle hypoattenuation within the cerebellar region (arrow), concerning for early ischemic changes in the posterior circulation. Early findings may be subtle and require high clinical suspicion, particularly in patients presenting with non-localizing neurologic deficits.

Emergent non-contrast computed tomography (CT) of the brain demonstrated no evidence of acute intracranial hemorrhage, mass effect, or midline shift. Subtle hypoattenuation within the posterior circulation territory was noted, raising concern for early ischemic changes (Figure 1). Given the high clinical suspicion for acute stroke, further vascular imaging was pursued.

**Figure 2:**
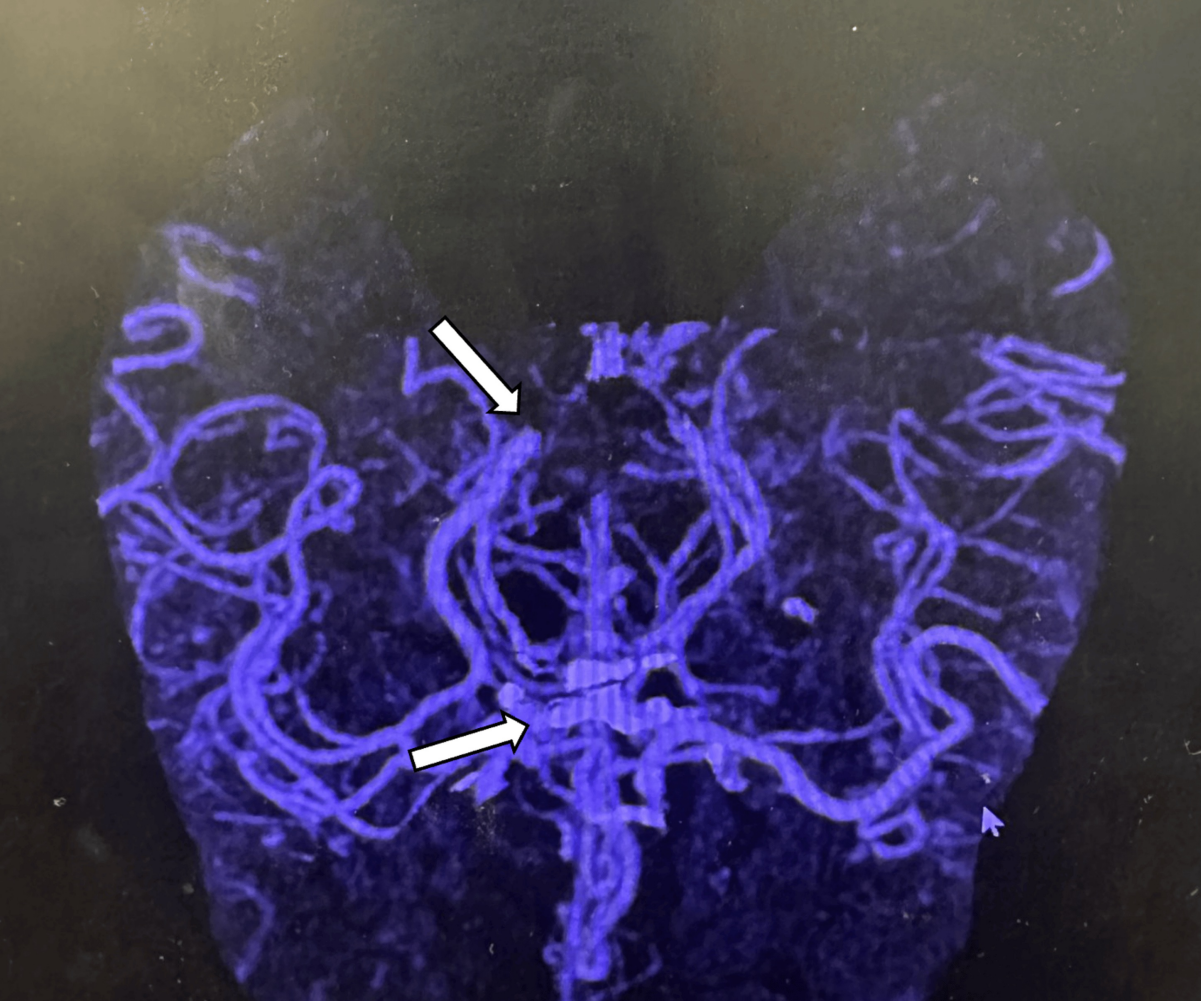
CT angiography demonstrating posterior circulation vascular anatomy. CT angiography of the head and neck demonstrates the vertebrobasilar circulation, highlighting high-grade stenosis of the basilar artery (arrow), consistent with the vascular findings described in the diagnostic evaluation.

CT angiography (CTA) of the head and neck confirmed high-grade basilar artery stenosis, corresponding to the vascular findings illustrated in Figure 2, without additional large vessel occlusion.

**Figure 3:**
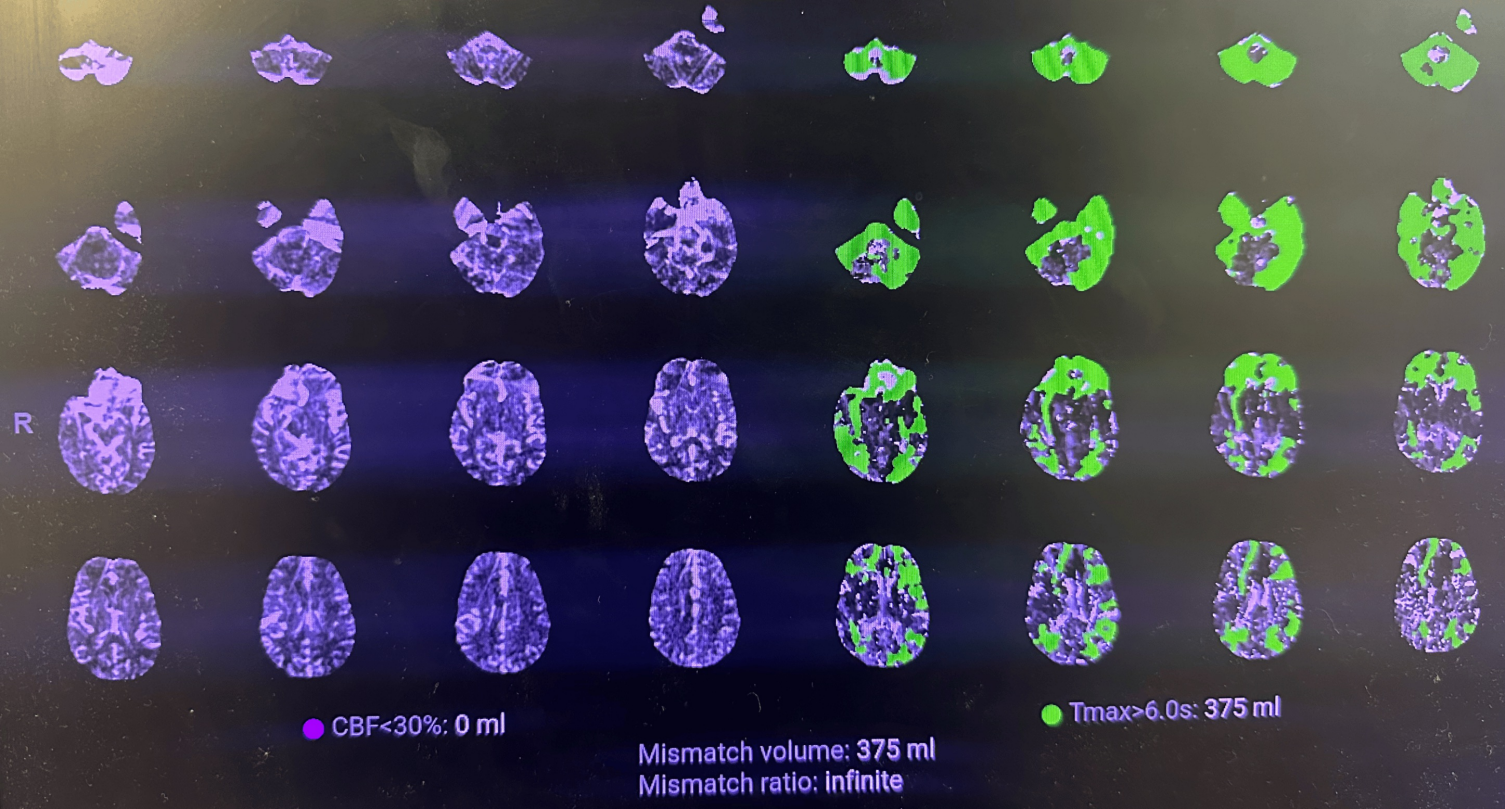
CT perfusion imaging demonstrating large perfusion mismatch in posterior circulation stroke. Multimodal CT perfusion maps demonstrate a large region of delayed perfusion (Tmax >6 seconds, green) involving bilateral cerebral and cerebellar hemispheres (arrows), without a corresponding reduction in cerebral blood flow (CBF <30%), indicating absence of a defined infarct core. This mismatch profile reflects a substantial volume of hypoperfused yet potentially salvageable tissue, supporting a physiology-driven approach to reperfusion therapy.

CT perfusion imaging demonstrated a large perfusion mismatch with no measurable infarct core and a substantial volume of hypoperfused, potentially salvageable tissue (Figure 3). The presence of a significant perfusion mismatch, in the absence of a defined infarct core, was the most critical imaging finding guiding management, as it indicated substantial salvageable tissue and supported escalation to thrombolysis and endovascular evaluation despite initially subtle findings on non-contrast CT.

**Figure 4:**
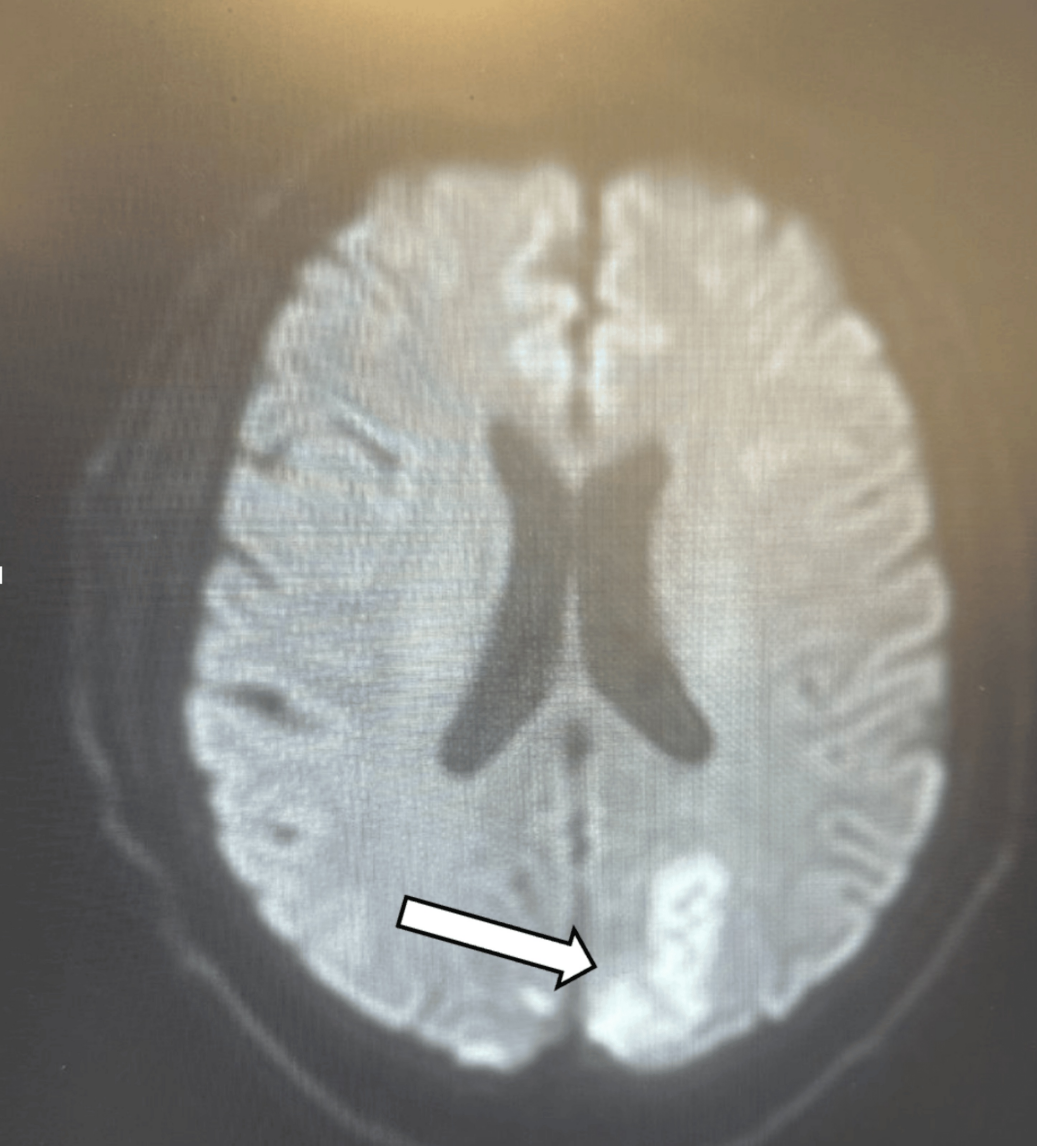
MRI brain demonstrating acute posterior circulation infarction. Axial diffusion-weighted imaging (DWI) demonstrates a focal hyperintense signal within the cerebellum (arrow), consistent with restricted diffusion and acute ischemia. These findings correlate with the patient’s presenting neurologic deficits and confirm posterior circulation infarction.

Magnetic resonance imaging (MRI) of the brain obtained during hospitalization confirmed an acute left cerebellar infarction, along with chronic infarcts involving the right pontine region and left thalamus, without hemorrhagic transformation (Figure 4).

Key imaging findings are illustrated in Figures 1-4, with arrows highlighting areas of early ischemia, vascular pathology, and perfusion mismatch.

The electrocardiogram showed normal sinus rhythm with possible left atrial enlargement and low-voltage QRS complexes, without evidence of acute ischemia.

Laboratory evaluation was notable for leukocytosis (WBC 12.1 × 10³/µL), mild anemia (hemoglobin 12.7 g/dL), severe hyperglycemia (glucose 538 mg/dL), and acute kidney injury, with creatinine rising from 1.60 mg/dL to a peak of 2.60 mg/dL during hospitalization. A comprehensive summary of laboratory findings is presented in Table 1. 

**Table 1:** Summary of laboratory findings at presentation and during hospitalization. Table 1 demonstrates severe hyperglycemia on admission, progressive acute kidney injury, and metabolic derangements, including low bicarbonate and electrolyte abnormalities. WBC: white blood cell count; Hgb: hemoglobin; BUN: blood urea nitrogen; eGFR: estimated glomerular filtration rate; CKD-EPI: Chronic Kidney Disease Epidemiology Collaboration; CO₂: bicarbonate; AST: aspartate aminotransferase; ALT: alanine aminotransferase.

Laboratory test	At presentation	During hospitalization	Reference range (units)
Glucose	538	137-174	70-100 mg/dL
Sodium	136	138-140	135-145 mmol/L
Potassium	3.9	3.3-3.6	3.5-5.0 mmol/L
Chloride	111	109-113	98-107 mmol/L
Bicarbonate (CO₂)	17	19-20	22-28 mmol/L
Anion gap	8	8-9	8-16
Serum osmolality (calculated)	285	297-305	275-295 mOsm/kg
Blood urea nitrogen (BUN)	15	31-46	7-20 mg/dL
Creatinine	1.60	2.30-2.60	0.7-1.3 mg/dL
eGFR (CKD-EPI)	50	28-33	>60 mL/min/1.73 m²
Calcium	7.3	7.5-7.6	8.6-10.2 mg/dL
Total protein	5.4	5.3	6.0-8.3 g/dL
Albumin	2.4	2.5	3.5-5.0 g/dL
Total bilirubin	0.5	0.3	0.1-1.2 mg/dL
AST (SGOT)	63	50	10-40 U/L
ALT (SGPT)	41	26	7-56 U/L
Alkaline phosphatase	94	116	44-147 U/L
Magnesium	1.8	2.4	1.7-2.2 mg/dL
Phosphorus	3.2	-	2.5-4.5 mg/dL

The observed metabolic and laboratory abnormalities were not incidental but likely contributed to the patient’s neurologic vulnerability and clinical trajectory. Severe hyperglycemia on presentation is known to exacerbate ischemic injury through increased lactic acidosis, oxidative stress, and disruption of the blood-brain barrier, potentially amplifying infarct progression despite preserved perfusion. Concurrent metabolic derangements, including low bicarbonate and electrolyte abnormalities, further reflect systemic stress and impaired physiologic homeostasis, which may compromise neuronal resilience in the setting of acute ischemia. Additionally, the development of acute kidney injury, likely multifactorial in the context of contrast exposure and critical illness, introduces important therapeutic and prognostic implications, including altered medication clearance and heightened susceptibility to further systemic complications. Collectively, these findings reinforce that posterior circulation stroke evolves within a dynamic physiologic environment, where metabolic instability and end-organ dysfunction may independently influence neurologic injury and recovery, supporting a systems-based framework of disease progression.

Multidisciplinary discussion

This case highlights posterior circulation stroke as a time-sensitive systems failure, in which neurologic injury reflects the interaction of vascular occlusion and rapidly evolving systemic complications.

From a neurology and neurointerventional perspective, BAO represents a neurologic emergency with a narrow window for meaningful recovery. The presence of a perfusion mismatch despite minimal early infarction underscores that tissue viability, rather than time alone, should guide reperfusion strategies, supporting rapid escalation to thrombolysis and endovascular evaluation.

From a critical care standpoint, management required airway protection, ventilatory support, and careful hemodynamic control to preserve cerebral perfusion while minimizing secondary injury. This phase emphasizes that outcomes are influenced not only by reperfusion but also by the quality of post-intervention physiologic support.

From an infectious disease and pulmonary perspective, the development of aspiration pneumonia reflects a common but high-impact complication of brainstem dysfunction. Prompt recognition and empiric antimicrobial therapy were essential to prevent further systemic deterioration.

From a nephrology and internal medicine standpoint, acute kidney injury - likely multifactorial in the setting of contrast exposure and critical illness - required close monitoring and conservative management to avoid further renal compromise. Concurrent metabolic derangements, particularly severe hyperglycemia, necessitated targeted intervention given their association with worsened neurologic outcomes.

An ophthalmology consultation identified a conjunctival lesion consistent with a probable pyogenic granuloma, managed conservatively, highlighting the importance of comprehensive evaluation in complex hospitalized patients.

Collectively, this case demonstrates that posterior circulation stroke is a multisystem emergency, in which outcomes depend on rapid diagnosis, imaging-guided intervention, and tightly coordinated multidisciplinary care.

Treatment

Given the imaging findings and clinical presentation concerning BAO, the patient was emergently transferred to a tertiary care center for advanced neurointerventional management. He received intravenous thrombolysis (tPA) followed by endovascular evaluation with digital subtraction angiography (DSA) targeting the posterior circulation. While formal reperfusion grading (e.g., Thrombolysis in Cerebral Infarction (TICI) score) was not documented, procedural reports indicated successful restoration of flow within the basilar artery, consistent with effective recanalization.

The patient required admission to the intensive care unit for close neurologic and hemodynamic monitoring. He was intubated for airway protection during the acute phase and remained mechanically ventilated for a limited duration in the intensive care unit, with subsequent successful extubation following neurologic and respiratory stabilization.

Secondary stroke prevention measures were initiated, including dual antiplatelet therapy (aspirin and clopidogrel) and high-intensity statin therapy. Blood pressure was managed with intravenous antihypertensive agents, including labetalol and hydralazine, and later transitioned to oral therapy.

The hospital course was complicated by aspiration pneumonia, treated with empiric broad-spectrum antibiotics (piperacillin-tazobactam), as well as acute kidney injury, which was managed conservatively with supportive care and nephrology consultation. Severe hyperglycemia was addressed with insulin therapy and close glucose monitoring.

The patient’s clinical trajectory reflects the cumulative impact of timely, coordinated interventions. Early administration of intravenous thrombolysis, followed by endovascular reperfusion, likely limited infarct progression by restoring perfusion to viable tissue, as suggested by the initial perfusion mismatch. Intensive care management, including airway protection and hemodynamic optimization, contributed to neurologic stabilization and prevention of secondary injury. The prompt recognition and treatment of aspiration pneumonia with antimicrobial therapy mitigated further systemic deterioration, while targeted management of hyperglycemia and metabolic derangements supported physiologic recovery. Additionally, conservative management of acute kidney injury helped prevent further end-organ dysfunction. Collectively, these interventions highlight how coordinated, systems-based care directly influenced both neurologic recovery and overall clinical outcome.

Clinical outcome 

The patient demonstrated gradual neurologic improvement over the course of hospitalization. At discharge, he was awake, alert, and oriented, with persistent but stable left-sided weakness consistent with prior deficits.

Renal function improved toward baseline, and infectious complications resolved with appropriate therapy. The patient remained hemodynamically stable and afebrile at the time of discharge.

He was discharged on dual antiplatelet therapy, statin therapy, antihypertensive medications, and an optimized insulin regimen, with plans for close outpatient follow-up with neurology, primary care, and endocrinology. He was also referred for continued rehabilitation to address residual functional deficits.

## Discussion

Background 

After the first description of a posterior circulation stroke due to basilar artery occlusion (BAO) by John Abercrombie, physicians throughout the 19th-century have referenced his work to continuously refine their understanding of BAOs [1]. Nowadays, BAOs are estimated to have an incidence of approximately one per 100,000 per year, which accounts for about 1% of all strokes that occur, with the most frequent cause being atherosclerotic occlusions (26-36%), followed by embolic occlusions from large artery and cardiac sources (30-35%) [1].

The risk factors for a posterior circulation stroke and BAO are the same as those for anterior circulation strokes, with certain differences; those differences include hypertension, diabetes mellitus, and metabolic syndromes [5]. Other differences that are more prominent in posterior circulation strokes and BAOs are those of race and sex differences; intracranial large-artery atherosclerosis is more common in African Americans, Asians, and women while atherosclerosis, that originates where the vertebral artery branches from the subclavian arteries, occur more in Caucasian men [6] The ANGEL-ACT registry showed that men represent about 79.3% of acute vertebrobasilar occlusion in patients that are undergoing endovascular therapy, nearly four times higher than women [7]. Other causes, but rare, include giant-cell arteritis, cervical trauma, upper cervical instability with rheumatoid arthritis, infectious arteritis, meningitis, aneurysms, and dilatative arteriopathies [1].

BAOs can be classified based on location along the vessel, which includes proximal, middle, or distal segments [1]. Proximal and middle segment occlusions cause a large pontine stroke with quadriplegia, while distal segment occlusions cause “top of the basilar syndrome” that includes symptoms of visual, oculomotor, and behavioral abnormalities [1].

Physiology and pathophysiology

BAO is a rare and serious form of large-vessel occlusion. It is an ischemic stroke with mortality rates reaching up to 80-90% without recanalization [8]. While ischemic strokes involving large- and medium-sized vessels in the anterior circulation often result from thromboembolism (such as cardiac or embolic strokes of unknown source), atherosclerotic disease with local intracranial stenosis more commonly causes BAO [9]. This case illustrates a posterior ischemic stroke caused by basilar artery stenosis and occlusion, probably related to underlying intracranial atherosclerosis and other patient comorbidities like diabetes mellitus, hypertension, and previous cerebrovascular stroke. The shift from hypoperfusion to infarction likely resulted from ischemia, which progressed as ATP depletion, failure of ionic pumps, calcium influx, and oxidative damage, eventually leading to the acute cerebellar infarct observed on his MRI. Early lesion development begins with lipoprotein accumulation in the intima, where oxidative changes and cytokine release promote the expression of adhesion molecules and chemokines [10]. Monocytes then infiltrate the arterial wall, transform into foam cells, and release more cytokines, oxidants, and matrix metalloproteinases, which cause the smooth muscle cells to migrate into the intima, proliferate, and produce extracellular matrix, leading to the formation of a lipid core and a fibrous cap [10]. Initially, the lesion enlarges outward, but as it grows, it narrows the lumen, resulting in stenosis [10].

Comparative analysis with current literature (clinical presentation, diagnostic workup, treatment, management, clinical outcome) 

Clinical Presentation

This 56-year-old male presented with altered mental status, dysarthria, and focal motor deficits, consistent with BAO, a condition characterized by heterogeneous and often non-localizing neurologic findings. Posterior circulation stroke frequently presents with fluctuating symptoms, contributing to delayed recognition and diagnostic uncertainty [1,11]. His moderate stroke severity (NIHSS 8) falls slightly below the NIHSS ≥10 threshold emphasized in recent randomized trials, including ATTENTION and BAOCHE, where endovascular therapy has demonstrated the greatest benefit [3,4].

Despite this, clinical severity in BAO does not always correlate with infarct burden, and patients with moderate NIHSS scores may still harbor large regions of salvageable tissue [11,12]. This highlights a key limitation of clinical assessment alone and reinforces the need for early imaging-based evaluation.

Diagnostic Workup

The diagnostic strategy in this case reflects current best practices, incorporating non-contrast CT, CT angiography, and CT perfusion imaging to rapidly identify vascular occlusion and tissue viability. Contemporary guidelines emphasize confirmation of BAO on CTA and the use of imaging-based metrics to guide intervention [11,13].

The presence of a significant perfusion mismatch in this patient supports a physiology-driven approach, in which treatment decisions are guided by salvageable tissue rather than rigid time thresholds [3,12]. This paradigm has become central in posterior circulation stroke, where early imaging may underestimate ischemic burden, and clinical deterioration can be rapid.

MRI findings of acute cerebellar infarction further illustrate the multifocal nature of BAO-related ischemia, which has been well described in prior literature [1]. These findings underscore the importance of advanced imaging in accurately defining disease extent and guiding timely intervention.

Management

Management with intravenous thrombolysis followed by endovascular thrombectomy is consistent with current standards of care. Randomized trials have demonstrated that thrombectomy significantly improves functional outcomes compared to medical therapy alone. The ATTENTION trial showed higher rates of functional independence with intervention, while the BAOCHE trial extended this benefit to patients presenting within a 4.5-24 hour window [3,4].

Meta-analytic data further support these findings, demonstrating improved outcomes across diverse patient populations and reinforcing the role of endovascular therapy as a cornerstone of BAO management [14]. Additionally, evidence suggests that bridging thrombolysis may confer additional benefit compared with thrombectomy alone in selected patients [14].

The use of dual antiplatelet therapy in this case represents a deviation from guideline-based recommendations. While dual therapy is supported in minor stroke and high-risk transient ischemic attack, its role in moderate-to-severe stroke remains uncertain, with current literature favoring single antiplatelet therapy for long-term secondary prevention [15,16]. In this case, the decision to initiate dual antiplatelet therapy was likely influenced by the presence of high-grade basilar artery stenosis, suggesting an underlying intracranial atherosclerotic process. In such contexts, dual antiplatelet therapy may be considered to reduce the risk of early re-occlusion and recurrent ischemic events, particularly following acute reperfusion strategies [14]. Additionally, the patient’s significant vascular risk profile, including diabetes mellitus, hypertension, and prior cerebrovascular disease, further supports a more aggressive antiplatelet approach, given the elevated risk of recurrent ischemic events in patients with intracranial atherosclerotic disease [11,16]. While current guidelines favor single antiplatelet therapy for long-term secondary prevention in moderate-to-severe stroke [15,16], this case highlights how individualized, pathology-driven decision-making may justify deviation from standard recommendations in selected high-risk patients.

Clinical Outcome

The patient’s gradual neurologic improvement represents a favorable outcome relative to historical BAO data, which demonstrate high rates of disability and mortality. Outcomes in BAO are strongly influenced by timely reperfusion, baseline stroke severity, and successful recanalization [3,11].

Importantly, this case highlights the contribution of systemic complications to the clinical trajectory. Aspiration pneumonia is a well-recognized complication of posterior circulation stroke, reflecting impaired airway protection due to brainstem involvement [11]. Acute kidney injury may also occur in the setting of contrast exposure and critical illness, further contributing to morbidity [17].

Prognostic factors associated with improved outcomes include lower NIHSS score, favorable imaging profile, successful reperfusion, and younger age, all of which were present in this case [11,13,16].

What we learned from this case 

This case supports the conceptualization of posterior circulation stroke as a time-sensitive systems-based process, in which clinical outcomes are influenced not solely by vascular occlusion, but by the interplay between diagnostic uncertainty, tissue-level physiology, and evolving systemic complications. Unlike anterior circulation events, posterior strokes often present with subtle or non-localizing findings, creating a critical window during which clinically significant ischemia may be underrecognized. In this context, the identification of a substantial perfusion mismatch despite minimal early infarction reinforces a key principle: tissue viability, rather than time alone, should guide therapeutic urgency and decision making.

Beyond initial diagnosis, this case highlights that neurologic injury frequently evolves within a broader physiologic environment. Brainstem dysfunction may impair airway protection and autonomic regulation, predisposing patients to complications such as aspiration, metabolic instability, and end-organ stress. Concurrent factors, including severe hyperglycemia and acute kidney injury, may further exacerbate neuronal vulnerability and influence recovery, underscoring the importance of integrated physiologic management.

Collectively, this case illustrates how coordinated, systems-based care, encompassing early imaging-guided intervention, proactive management of secondary complications, and multidisciplinary collaboration, can directly influence both neurologic and systemic outcomes. While derived from a single case, this framework highlights a clinically relevant perspective that may inform future investigation and guide more holistic approaches to posterior circulation stroke management.

## Conclusions

This case illustrates a systems-based perspective for understanding posterior circulation stroke as a time-sensitive process, in which neurologic outcomes are shaped not only by timely reperfusion, but also by the recognition and management of secondary physiologic insults. The presence of a substantial perfusion mismatch despite minimal early infarction reinforces the importance of physiology-driven decision-making, while the patient’s clinical course highlights how complications such as aspiration pneumonia, metabolic instability, and acute kidney injury may influence disease trajectory.

Accordingly, optimal management extends beyond isolated, time-based intervention to encompass coordinated care that integrates early diagnostic clarity with proactive, multidisciplinary management of systemic complications. While the concept of posterior circulation stroke as a “systems-based” or “systems-level” process is supported by the clinical course described, it should be interpreted as a conceptual and hypothesis-generating framework rather than a definitive paradigm. Several elements of this approach - including early recognition of atypical presentations, rapid use of multimodal imaging to assess tissue viability, and anticipatory management of complications - represent reproducible principles that may be broadly applicable in clinical practice. Further investigation in larger studies is warranted to better define the role of systems-based approaches in posterior circulation stroke management.
